# Impact of the Potential m^6^A Modification Sites at the 3′UTR of Alfalfa Mosaic Virus RNA3 in the Viral Infection

**DOI:** 10.3390/v14081718

**Published:** 2022-08-04

**Authors:** Luis Alvarado-Marchena, Mireya Martínez-Pérez, Jesús R. Úbeda, Vicente Pallas, Frederic Aparicio

**Affiliations:** 1 Instituto de Biología Molecular y Celular de Plantas, Consejo Superior de Investigaciones Científicas—Universitat Politècnica de València, Avda. Ingeniero Fausto Elio, 46022 Valencia, Spain; 2 Centro de Edafología y Biología Aplicada del Segura (CEBAS)-CSIC, Departamento de Biología del Estrés y Patología Vegetal, 30100 Murcia, Spain

**Keywords:** *N*^6^-methyladenosine, RNA covalent modifications, plant alfamovirus, DRACH motif, in vivo AMV replication, 3′UTR

## Abstract

We have previously reported the presence of m^6^A in the AMV (Alfamovirus, *Bromoviridae*) genome. Interestingly, two of these putative m^6^A-sites are in hairpin (hp) structures in the 3’UTR of the viral RNA3. One site (_2012_AAACU_2016_) is in the loop of hpB, within the coat protein binding site 1 (CPB1), while the other (_1900_UGACC_1904_) is in the lower stem of hpE, a loop previously associated with AMV negative-strand RNA synthesis. In this work, we have performed in vivo experiments to assess the role of these two regions, containing the putative m^6^A-sites in the AMV cycle, by introducing compensatory point mutations to interfere with or abolish the m^6^A-tag of these sites. Our results suggest that the loop of hpB could be involved in viral replication/accumulation. Meanwhile, in the _1900_UGACC_1904_ motif of the hpE, the maintenance of the adenosine residue and the lower stem hpE structure are necessary for in vivo plus-strand accumulation. These results extend our understanding of the requirements for hpE in the AMV infection cycle, indicating that both the residue identity and the base-pairing capacity in this structure are essential for viral accumulation.

## 1. Introduction

Alfalfa mosaic virus (AMV) is the only member of the Alfamovirus genus in the *Bromoviridae* family [1]. AMV presents a tripartite single-stranded RNA genome of messenger-sense polarity that is capped (m^7^G) at the 5′ end and lacks poly A tail at the 3′ terminus. RNAs 1 and 2 encode the replicase subunits P1 and P2, respectively, whereas RNA 3 encodes the movement protein (MP) and serves as a template for the synthesis of the subgenomic RNA 4 (sgRNA 4), which encodes the coat protein (CP) [2]. Like ilarviruses, AMV requires the presence of the CP to initiate infection [3]. The terminal 145 residues in the 3′ untranslated regions (3′UTR) is > 80% homologous in the three AMV RNAs and can fold into a similar secondary structure, consisting in a linear array of stem-loop structures, flanked by AUGC motifs [4], which contains several independent high-affinity binding sites for CP [5]. Thus, by in vitro binding assays, it was found that hairpins A and B (hpA and hpB) and the flanking AUGC motif 1, 2 and 3 represent the minimal CP binding site 1 (CPB1), whereas hairpins F and G (hpF and hpG) and AUGC motifs 4 and 5 conform the CPB2 [6]. The binding of the CP to the 3′UTR is critical for AMV to initiate infection and stimulates the translation of AMV RNAs, most probably by mimicking the function of the poly(A)-binding protein [7,8]. Moreover, CP is a component of the AMV replicase [9]. Besides the binding sites for the CP, hairpin E (hpE) was found to be essential for minus-strand synthesis in vitro [10].

*N*^6^-methyladenosine (m^6^A) is a widespread modification on cellular RNAs of different organisms, including the genomes of some viruses that are dynamically regulated, and can impact many cellular processes and pathways [11,12,13,14]. In plants, m^6^A methylation is mainly installed by a methylation complex containing several proteins, as follows: mRNA adenosine methylase A (MTA), MTB, FKBP12 INTERACTING PROTEIN 37KD (FIP37), VIRILIZER (VIR) and HAKAI. Most recently, FIONA1 (FIO1), a human METTL16 ortholog, was also described as a m^6^A methyltransferase that modulates floral transition in arabidopsis [12,15,16]. Moreover, this modification is removed by demethylases of the *AlkB* family [17,18], and members of the EVOLUTIONARILY CONSERVED C-TERMINAL REGIONS (ECT) family are the best-described proteins that recognize and process m^6^A-modified RNAs [19]. In arabidopsis, m^6^A controls plant development at the embryonic stage, vegetative growth and flowering [19]. Remarkably, diverse studies have demonstrated that, in addition to its involvement in physiological processes, the m^6^A pathway also modulates viral infections in mammals [20]. 

We previously reported the presence of m^6^A along the RNAs of AMV, and that suppression of the m^6^A demethylase *Arabidopsis* protein ALKBH9B increases the abundance of m^6^A on the viral genome. Furthermore, the systemic infection capability of the virus was clearly reduced in *alkbh9b* plants, nearly blocking floral stem invasion [21,22]. Additionally, by a methylated-RNA immunoprecipitation sequencing experiment, we mapped several discrete peaks that were distributed along the AMV genome, which were susceptible to be m^6^A methylated. Two of these sites were located in two hairpin structures in the 3′UTR of the genomic RNA 3, one of them was within the CPB1, in the loop of hpB, whereas the other was located in the lower stem of hpE.

Previous studies have shown that the structural integrity of hpB and hpE on RNA 3 is essential for the AMV replication cycle, through their role in CP binding and minus- strand synthesis, respectively [2]. Moreover, it has been found that m^6^A methylation can affect A-U base pairing, which may alter putative RNA–protein interactions [23,24]. Although the impact of m^6^A on RNA structure and function has been clearly demonstrated in animal viruses, its significance has yet to be firmly established in the case of plant RNA viruses [12]. Thus, we carried out in vivo experiments to evaluate the roles of these two putative m^6^A-sites in the AMV cycle by introducing compensatory point mutations to interfere with or abolish the m^6^A methylation of these sites.

## 2. Materials and Methods 

### 2.1. Viral Constructs

**Figure 1:**
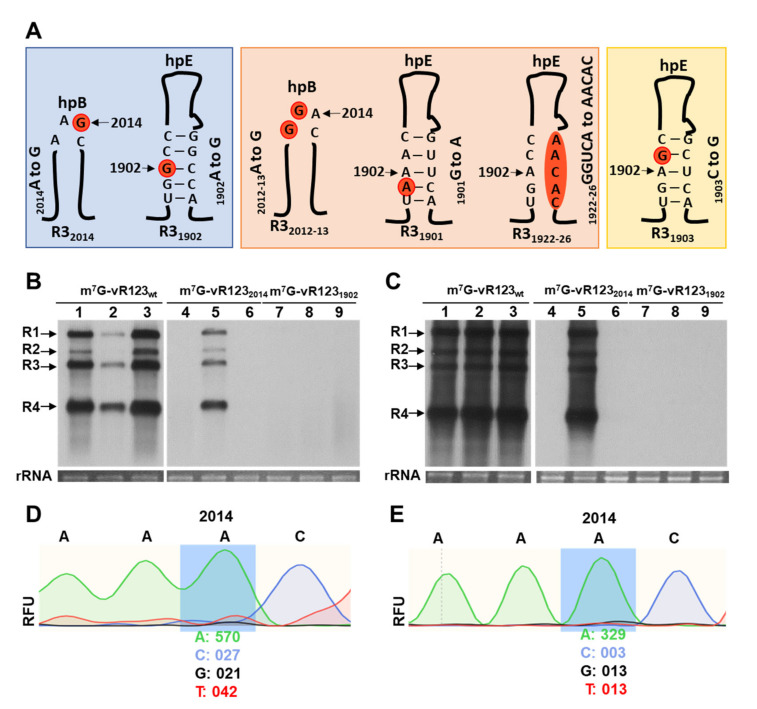
Schematic representation of the different mutations on hpB and hpE structures of the 3-UTR in the RNA 3 analyzed in this study and effect of mutations in DRACH motives in the 3′UTR-RNA 3, using RNA transcript inoculation procedure. (**A**): Mutant names are indicated at the bottom of each mutant. Mutated nucleotides are indicated in orange circles. In blue shading, mutant RNAs with the potential methylated A is substituted to G. In orange shading, RNAs with mutations around this A, but keeping a DRACH motif. In yellow shading, mutant RNAs in which A is conserved but DRACH is disturbed. (**B**,**C**): Representative Northern blots assays from inoculated (**B**) and upper non-inoculated leaves (**C**) of *N. benthamiana* plants at 6- and 13- days post-inoculation, respectively. Three plants were mechanically inoculated with different mixtures of the three RNAs plus CP (indicated at the top of the panels). Positions of the vRNAs are indicated on the left. Ethidium bromide staining of ribosomal RNAs was used as RNA loading control. (**D**,**E**): Representative electropherogram of the _2012_DRAC_2015_-motif, located in the hpB of the 3′UTR-RNA 3 in inoculated (**D**) and upper non-inoculated leaves (**E**) with m^7^G-vRNA123_2014_. RFU values (relative fluorescence units) of the A and G nucleotides are indicated at the bottom of the mutated position.

For infection with viral transcripts, RNA 1, 2 and 3 of the AMV PV0196 isolate (Plant Virus Collection, DSMZ) were cloned into pTZ57R/T (Thermo Scientific™, Waltham, MA, USA), generating the plasmids ptZ/cDNA1, ptZ/cDNA2 and ptZ/cDNA3. Using ptZ/cDNA3, mutagenic PCRs was performed to disable the putative DRACH sites, located at loop hpB and stem hpE. Thus, specific primers (Table A1) were designed with point mutations to change the following: (i) the putative _2014_m^6^A residue in loop hpB for a guanosine residue (A to G), (ii) the putative _1902_m^6^A in the lower stem hpE for cytosine (A to C) and (iii) the _1903_C residue next to the _1902_m^6^A for a guanosine (C to G). Compensatory mutations were also introduced to preserve the hairpin structure in mutations located at positions 1902 and 1903 (Figure 1 and Table A1). After the treatment with Esp3I and DpnI endonucleases to digest the restriction sites and the parental DNA template, PCR products were ligated and transformed in *Escherichia coli* DH5α cells and mutation-containing plasmids. ptZ/cDNA3 mutants (ptZ/cDNA3_2014_; ptZ/cDNA3_1902_ and ptZ/cDNA3_1903_) were confirmed by sequencing. Next, 300 ng of each plasmid that composes the viral RNA of AMV were linearized with PstI and transcribed with T7 RNA polymerase (Takara^TM^, Shiga, Japan). Viral RNAs (vRNAs) were m^7^G-capped (m^7^G-vRNAs) using ScriptcapTM m^7^G Capping System (Epicentre^®^ Biotechnologies, Madison, WI, USA), according to the manufacturer’s instructions.

For infection with AMV/PV0196-infectious clone, RNA 1, 2 and 3 were cloned into a pBluescript SK(+) (Addgene^®^, Watertown, MA USA), as follows: pSK/cDNA1, pSK/cDNA2 and pSK/cDNA3. The cDNAs were inserted between the cauliflower mosaic virus (CaMV) 35S promoter and the hepatitis delta virus (HDV) ribozyme sequence, since the inclusion of this ribozyme at the end of the viral cDNAs was previously shown to enhance their infectivity [25]. To obtain the mutant pSK/cDNA3, the procedure described above was carried out. Additionally, three new mutations were developed in the following: (i) the _2012_A_2013_A residues next to the _2014_A for guanosines (AA to GG), (ii) the _1901_G residue next to the _1902_A for an adenosine (G to A) and (iii) the _1922_GGUCA_1926_ residues in lower stem hpE for AACAC nucleosides. The mutation located at position 1901 required another compensatory mutation to preserve the hairpin structure (Figure 1 and Table A1). pSK/cDNA3 mutants (pSK/cDNA3_2014_; pSK/cDNA3_1902_; pSK/cDNA3_2012-13_; pSK/cDNA3_1903_; pSK/cDNA3_1901_; and pSK/cDNA3_1922-26_) were confirmed by sequencing. Finally, each expression cassette of the plasmid pSK [35S::RNA1::Rz::PoPit], [35S::RNA2::Rz::PoPit] and pSK [35S::RNA3_wt or mutant_::Rz::PoPit] was introduced into the pMOG800 binary vector using a unique HindIII restriction site. Next, all binary vectors were transformed into *Agrobacterium tumefaciens* C58 cells.

### 2.2. Infection and Viral RNA Analysis

Infectious transcripts and cDNAs were evaluated in 2-week-old *Nicotiana benthamiana* plants. The AMV m^7^G-vRNAs were mechanically inoculated with a mixture of 1 ug of m^7^G-RNA1 and m^7^G-RNA2, and the corresponding m^7^G-RNA3 (wild-type or mutants), plus 1.9 μg of AMV 6XHis—CP (previously described in [21]) in 30 mM of sodium phosphate buffer, pH 7.0. Three biological replicates were performed, every replicate consisted in three *N. benthamiana* plants inoculated with each m^7^G-vRNA123 combination. Meanwhile, for the agroinfiltration infections, AMV infectious clones were mixed at an optical density at 600 nm of 0.025 each, in infiltration solution (10 mM MES, pH 5.5 and 10 mM MgCl_2_) and infiltrated (three plants with each pMOG/cDNA123 combination). In all experiments, total RNA was extracted using EXTRAzol. The detection of viral RNAs (vRNAs) was carried out by Northern blot analysis, as previously described [26], using a digoxigenin-labeled probe to detect the multipartite genome of AMV (DigAMV). Viral RNA detection was conducted using CSPD chemiluminescent substrate. Densitometry was performed using ImageJ 1.53c (Wayne Rasban, National Institutes of Health). Statistical significances at the 95% confidence level (α = 0.05) were determined using Minitab 18 (*p* < 0.05), through one-way analysis of variance and Fisher’s least significant difference (LSD) test for multiple comparisons.

To analyze putative reversions of the introduced mutations, the 3′UTR AMV RNA 3 from all infected plants was amplified by RT-PC, using specific primers. PCR products were separated in agarose gels and the correct size products were purified and directly sequenced by Sanger sequencing method, with an ABI 3130 XL capillary sequencer quantitative PCR at the DNA Sequencing Service of the IBMCP.

### 2.3. Electrophoretic Mobility Shift Assay (EMSA)

To evaluate if these mutations interfered with the RNA/CP interaction, we performed EMSA experiments. For this, the 3′UTR of the corresponding pSK/cDNA3′s plasmids were amplified using a forward primer located at the end of the CP open reading frame, containing the sequence of the T7 promoter, and a reverse primer located at the 3′ 20 last residues (Table A1). To perform EMSA experiments, PCR products were used as templates to synthetize plus-strand RNAs corresponding to the 3′UTR region of the RNA3 wild-type (wt) and mutants (3′UTR_wt_, 3′UTR_2014_, 3′UTR_1902_ and 3′UTR_2012-13_), which were incubated with increased concentrations of AMV CP, as described in [26]. For this, we expressed and purified the CP protein using an N-terminal histidine tag (6xHis-CP) in a bacterial system, as previously described in [21].

## 3. Results and Discussion

In a previous study, we mapped several discrete peaks that were distributed along the AMV genome, which were susceptible to be m^6^A methylated [21]. Some of the potentially methylated bases were located in the 3′UTR of the RNA 3, forming part of a canonical m^6^A motif DRACH (D = A, G or U, R = G/A, H = A/U/C) [23]. One site (_2012_AAACU_2016_) was positioned within the CPB1, in the loop of hpB, whereas the other (_1900_UGACC_1904_) was located in the lower stem of hpE (Supplementary Figure S1). In this work, we have analyzed their putative role in the AMV infection cycle by introducing point mutations that affect different key residues in both DRACH sites (Figure 1A). 

Thus, *N. benthamiana* leaves were inoculated with a mixture of capped transcripts of the RNA 1, RNA2 and RNA 3 wt or mutated versions of the potential m^6^A residues (Figure 1A, R3_2014_ and R3_1902_ mutants, blue shading) in the presence of the AMV CP. Northern blot assays showed that plants inoculated with wild-type m^7^G-vRNAs showed 100% infection effectiveness (Figure 1B,C: lanes m^7^G-vRNA123_wt_), which demonstrates that the inoculum was fully functional to initiate the infection cycle and perform systemic movement. Contrarily, we found that in plants inoculated with the mixture m^7^G-vR123_2014_, only one sample out of three accumulated viral RNAs (vRNAs) in the inoculated and non-inoculated leaves, whereas the inoculum m^7^G-vR123_1902_ failed to accumulate both in inoculated and upper non-inoculated leaves (Figure 1B,C: lanes m^7^G-vRNA123_2014_ and m^7^G-vRNA123_1902_). The same results were consistently observed in three independent trials (Table A2). Overall, these results indicate that the elimination of residue A in these DRACH sites interfered with the early stages of AMV replication. 

A nucleotide reversion at the mutated site could be the reason why one of three plants inoculated with m^7^G-vR123_2014_ inoculum resulted in local and systemic infection. To check this possibility, an RT-PCR was carried out with specific primers to amplify the 3′UTR of the RNA 3 from the total RNAs extracted from infected plants. The sequencing of the PCR product showed the nucleotide reversion of the G_2014_ mutation to A, in both inoculated and non-inoculated tissue (Figure 1D,E: relative fluorescence units—*RFU*—detected was 96.4% and 96.2% of A, compared to 3.6% and 3.8% of G).

As stated above, in the case of alfamovirus, inoculation with transcripts requires that vRNAs are capped and either RNA 4 or CP must be present in the inoculum. All these requirements could interfere with the effectiveness of the inoculation procedure (Figure 1B, lanes m^7^G-vRNA123_wt_). In addition, it is well known that *Agrobacterium*-based inoculation methods are much more efficient than transcript-based procedures ([27,28]). Thus, to circumvent the necessity of preparing in vitro transcripts and to avoid, as much as possible, the failures of the infection process, the three cDNAs of AMV were cloned into binary vectors, driven by the 35S promoter of cauliflower mosaic virus. 

**Figure 2:**
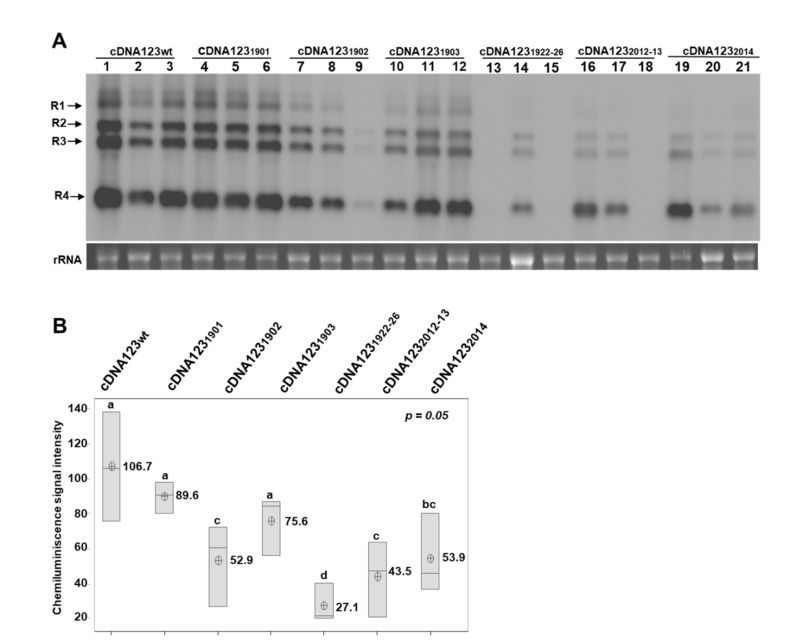
Effect of mutations in DRACH motifs of the 3′UTR-RNA 3, using the agroinfiltration procedure. (**A**): Representative Northern blot assays from upper non-infiltrated leaves of *N. benthamiana* plants, at 5 days post-agroinfiltration. Three plants were agroinfiltrated with different mixtures of cDNAs (indicated at the top of the panels). Positions of the vRNAs are indicated on the left. Ethidium bromide of ribosomal RNAs was used as RNA loading control. (**B**): Boxplots of the densitometric analysis of AMV vRNAs accumulation. The lower and upper limits of the boxes plot the min and max values, respectively, whereas the lines dividing them represent the median values. Points inside boxes represent the mean from the three replicates. *p* < 0.05 indicates statistical significance. Means that do not share a letter are significantly different.

To confirm the ability of the AMV cDNAs to initiate the infection cycle, a mixture of agrobacterium cells transformed with each binary vector was agroinfiltrated in *N. benthamiana* plants. This approach resulted in the infection of the three inoculated plants (Figure 2A, lanes cDNA123_wt_). Next, the infection efficiency of the R3_2014_ and R3_1902_ mutants (Figure 1A) was evaluated using this same approach. In contrast to the RNA inoculation procedure, agroinfiltration with the mixture cDNA123_2014_ rendered the infection of all agroinfiltrated plants, although with a reduction in viral accumulation of 49.5%, with respect to the wt virus (Figure 2A,B, cDNA123_2014_). Likewise, all plants agroinfiltrated with the mixture cDNA123_1902_ were infected, although the viral accumulation in upper non-inoculated leaves showed a reduction of 50.4% (Figure 2A,B, cDNA123_1902_). Overall, our results suggest that the identity of these residues is important for AMV infection. 

Regarding the DRACH motif in hpB structure, the upstream _2012_AA_2013_ residues were changed for G (Figure 1A, R3_2012-13_ mutant, orange background) and the Northern blot analysis of plants agroinfiltrated with this mutant showed a reduction of 59.2%, with respect to the wt virus (Figure 2A,B, cDNA123_2012-13_). Interestingly, the sequencing of the 3′UTR-RNA3 of the plants inoculated with cDNA123_2014_ or cDNA123_2012-13_ showed a total or partial reversion of the mutated nucleotides to wild-type sequence (Supplementary Figure S2). These results suggest that the reversion to A, in positions 2012 to 2014, would be linked with increased viral accumulation levels, indicating that it is an important structural requirement for in vivo viral replication and/or accumulation. Similar effects have been reported in dengue virus, in which point mutations in the upper loop of the hairpin A (SLA) of the 5’UTR were found to produce non-replicating RNAs, and nucleotide reversions within the SLA are sufficient to restore promoter activity [29]. Moreover, point changes in the third base of the stem-loop of the mouse histone H2a-614 gene have been shown to greatly reduce the expression of histone mRNA. A similar reduction was found in the ability to process the same mutant pre-mRNAs in vitro [30].

**Figure 3:**
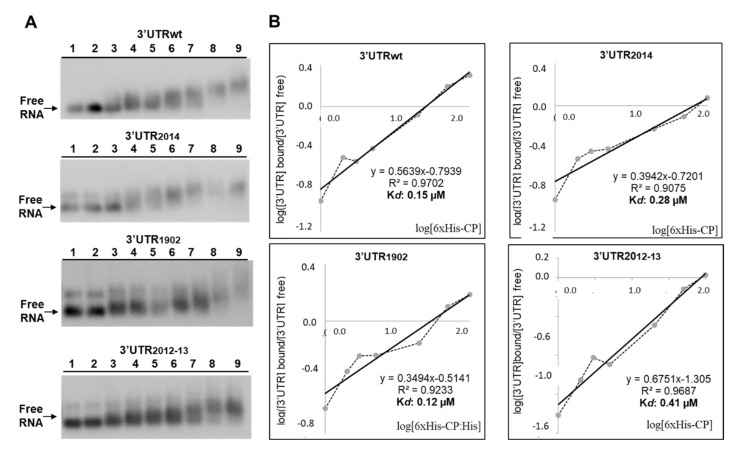
Analysis of RNA–protein complexes, formed between purified 6xHis-CP protein and the WT and mutated versions of the 3′UTR transcript of AMV-RNA. (**A**): Example of an EMSA after incubation with 5 ng of 3′UTRs transcript wt, or the indicated mutant without protein (lanes 1 and 2), or with 1, 2, 3, 5, 20, 50 and 100 ng of 6xHis-CP (lanes 3 to 9) corresponding to 0.004, 0.008, 0.01, 0.02, 0.08, 0.20 and 0.41 µM, respectively. (**B**): Representation of Hill transformation of the RNA-CP binding obtained from three independent experiments. R2 coefficient, regression equation and the dissociation constant (K*d*) are shown.

On the other hand, it is known that the binding of the CP to the CPB1 region in the RNA3 is critical to establish the AMV infection cycle [31]. For this reason, we evaluated whether the mutations on hpB_2014_ and hpB_2012-13_ were interfering with the interaction of the CP with the full-length 3′UTR of the AMV RNA3, which would explain the reduced infectivity of the inocula containing these mutated RNA3. Furthermore, in spite of the fact that hpE has never been found to be implicated in CP interactions, we included mutant hpE1902 in the experiment. The EMSA analysis showed a decrease in the free RNA at quantities exceeding 20 ng of 6xHis:CP, indicating the formation of protein–RNA complexes with all three 3′UTRs (Figure 3A). The apparent constant dissociation (*K_d_*) was calculated from the linear regression of the mean values from at least three technical replicates [32]. The *K_d_* value of CP was estimated to be 0.15 µM for 3′UTR_wt_, 0.28 µM for 3′UTR_2014_, 0.12 µM for 3′UTR_1902_ and 0.41 µM for 3′UTR_2012-13_ (Figure 3B). These *K_d_* values are similar to those previously reported for AMV CP (0.5 μM) [33] and for other plant virus proteins, such as the CP of turnip crinkle virus (0.5 μM) [34] and p7 MP of carnation mottle virus (0.7 μM) [32]. Previous studies reported that the loop of hpB would not disturb in vitro CP binding to the last 39 nt fragment of the 3′UTR [6]. Similarly, our results indicate that the DRACH sequence, located neither in the hpB loop nor in hpE, is involved in sequence-specific interactions with the CP. However, it is well known that the m^6^A-U base pair is weaker than the A-U base pair, which can lead to the destabilization of stem-loop structures and, consequently, can alter putative RNA–protein interactions (reviewed in Zaccara et al. [35]). Therefore, we cannot rule out the possibility that post-transcriptional modifications in this region could induce RNA structural changes that interfere with RNA–protein interactions.

Next, to evaluate if the observed effects in the R3_1902_ mutation were due to the m^6^A-site substitution or a structural alteration in this stem structure, we generated a series of mutants, maintaining the A residues susceptible to be methylated but preserving or altering the DRACH consensus site. Thus, _1901_G and _1903_C residues in hpE were changed for A and G, respectively (Figure 1A, R3_1901_ and R3_1903_, orange and yellow shading), and compensatory mutations were also introduced to preserve the hairpin structure in these mutants. The plants agroinfiltrated with both mutant cDNA123_1901_, which keeps the DRACH motif, and mutant cDNA123_1903_, in which the DRACH motif is disrupted, showed a not statistically significant reduction in viral accumulation, with respect to the wt virus (Figure 2A,B, cDNA123_1901_ and cDNA123_1903_). Furthermore, a sequence analysis showed that the mutations were maintained in all plants (Supplementary Figure S2). In contrast, as stated above, viral accumulation in plants agroinfiltrated with the mixture cDNA123_1902_ showed an overall reduction of 50.4%, with respect to the wt virus (Figure 2B, cDNA123_1902_). More interestingly, the samples with the highest viral accumulation (Figure 2A, cDNA123_1902_, lanes 7 and 8) had reverted the mutation to adenosine, whereas the sample with the faintest signal (Figure 2A, cDNA123_1902_, lane 8) presented a population of different mutations around the A_1902_ position (Supplementary Figure S2, R3_1902*_ sequence). Thus, although the identity of this residue seems to be critical for AMV infection, our results suggest that m^6^A methylation would not be essential in this case.

A previous study showed that hpE is a crucial element for AMV minus-strand in vitro synthesis [30]. Furthermore, the identity and base-pairing capability of the upper-stem (Supplementary Figure S1, just below of the UCG triloop) was essential in this process, whereas, when the lower stem—in which the DRACH sequence is located—was eliminated, minus-strand synthesis was reduced approximately 60% [30]. To evaluate the importance of the hpE structure for in vivo viral replication, _1922_GGUCA_1926_ residues in lower stem hpE were changed for AACAC residues, disrupting the base pairing (Figure 1A, mutant hpE_1922-26_, orange shading). As shown in Figure 2A, only one plant agroinfiltrated with this mutant showed vRNAs accumulation, which additionally presented a reduction of 74.6%, with respect to the wt virus (Figure 2B, cDNA123_1922-26_). Overall, our results showed that both _1902_A residue and that the base-pairing capability of the hpE lower stem are critical for AMV plus-strand in vivo synthesis. It has been proposed that hpE must consist of an interrupted 10 bp stem-base pairing to be functional [36]. Altering the base-pairing capability of the hpE in the cDNA3_1922-26_ mutant causes a reduction in stem size, a key structural requirement for hpE-promoting activity.

## 4. Conclusions

In this work we have evaluated the in vivo role of two DRACH motifs located in the hpB loop and hpE stem of the RNA 3 3′UTR that, interestingly, led us to discover the hot sites involved in the initiation of in vivo AMV replication. The reversion of the mutated nucleotides observed in the hpB_2014_ mutant indicates that the identity of the A residues—at positions 2012–2014, in loop hpB (_2012_AAACU_2016_)—represent a key structural requirement for in vivo viral replication. In this context, it is important to highlight that, as far as we know, this is the first time that the loop of hpB has been shown to be involved in viral replication/accumulation. Although the EMSA analysis showed that the mutations in the hpB loop did not alter the in vitro binding capacity to the CP, we cannot rule out the possibility that N6-adenosine methylation of this site would modify the hpB structural conformation, altering the in vivo binding of the CP or another viral or host proteins to this stem-loop. Interestingly, both _1902_A residue identity and the maintenance of the lower stem hpE structure at a 10 bp length stem are necessary to obtain wild-type plus-strand accumulation in vivo. These results extend our knowledge of the requirement of hpE in the AMV infection cycle, so that both the identity and base-pairing capability of bases in this structure are essential for minus-strand [30] and plus-strand synthesis.

## Data Availability

The original contributions presented in the study are included in the manuscript, further inquiries can be directed to the corresponding author.

## Supplementary Materials

The following supporting information can be downloaded at: https://www.mdpi.com/article/10.3390/v14081718/s1 Figure S1. Schematic representation of the strucutre and mutants in 3′UTR of the AMV RNA3 analyzed in this study. Linear secondary structure conformation proposed for the 3′UTR of RNA3. AUGC-sequence motifs and minimal CP-binding sites are underlined. Hairpins (hp) are labeled A to E. The nucleotides in the RNA 3 sequence are numerated from the first 5’ residue of the full-length RNA 3. Putative DRACH in hpB and hpE are indicated. Adenosine residues at postions 1902 and 2012 susceptible to be m6A modified are shown. Figure S2. Nucleotide alignments of 3’UTR regions in the RNA 3 containing the (_1900_UGACC_1904_) and (_2012_AAACU_2016_) sites (yellow shading, whereas the A residue susceptible to be methylated is underlined). Mutant names are indicated in the left. The 3’UTR region in the RNA 3 was amplified by RT-PCR from total RNA extracted of systemic leaves of plants agoinfiltrated with the different mutants. PCR products were directly sequenced. The average nucleotide sequence obtained for each mutant were aligned with CLUSTAL W program. In green and blue shading are shown non-reverted and reverted nucleotides, respectively. Compensatory mutations in (_1922_GGUCA_1926_) site to maintain lower hpE stem structure are shown. N = A/G; R = C/A; X = G/U; P = C/G.

## Appendix A

**Table A1:** Primers used in this study.

Primer	Sequence
ss/R3-3′UTR	5′- TAATACGACTCACTATAGACGATCTTGATCGTCAATGA -3′
as/R3-3′UTR	5′- TATAGTGAGTCGTATTAGCATCCCTTAGGGGCATTCA -3′
ss/mut_2014	5′- AGTCCGTCTCCTCATGCAAAGCTGCATGAATGC -3′
as/mut_2014	5′- AGTCCGTCTCCATGAGCATTTATATATGTGCGTTAG -3′
ss/mut_1902	5′- AGTCCGTCTCGGGTGGATTAAGGGCAAGGTATGAAGT -3′
as/mut_1902	5′- AGTCCGTCTCCCACCCAGTGGAGGGCAGCATTAAATGA -3′
ss/mut_2012-13	5′- ACTGCGTCTCGGACTGCATGAATGCCCCTAAG -3′
as/mut_2012-13	5′- AGTCCGTCTCCAGTCCTGCATGAGCATTTATATATGTGCGT -3′
ss/mut_1903	5′- AGTCCGTCTCGTGGATTAAGCTCAAGGTATGAAGTCCTATTCG -3′
as/mut_1903	5′- AGTCCGTCTCATCCACCCAGTGGAGCTCAGCATTAAAT -3′
ss/mut_1901	5′- AGTCCGTCTCACCTCCACTGGGTGGATTAAGGTTAAGG -3′
as/mut_1901	5′- AGTCCGTCTCGGAGGTTAGCATTAAATGACTTTAGCATCCC -3′
ss/mut_1922-26	5′- ACTGCGTCTCAACACAGGTATGAAGTCCTATTCGCTCC -3′
as/mut_1922-26	5′- AGTCCGTCTCTGTGTTTTAATCCACCCAGTGGAGGTCAG -3′

**Table A2:** Detection of AMV by Northern blot assays in three biological replicates performed in N. benthamiana, after inoculation with infectious transcripts (wild-type and mutants).

	Biological Replicate No. 1			Biological Replicate No. 2			Biological Replicate No. 3		
	Plant 1	Plant 2	Plant 3	Plant 1	Plant 2	Plant 3	Plant 1	Plant 2	Plant 3
m^7^G-vR123_wt_	+/+	+/+	+/+	+/+	+/+	+/+	+/+	+/+	+/+
m^7^G-vR123_2014_	+/+	−/−	−/−	−/−	+/+	−/−	+/+	−/−	−/−
m^7^G-vR123_1902_	−/−	−/−	−/−	−/−	−/−	−/−	−/−	−/−	−/−

+/+ AMV was detected in inoculated and systemic tissues. −/− AMV was neither detected in inoculated nor in systemic tissues. +/− AMV was detected in inoculated tissue but not in systemic tissues.
