# Supplementary figures and images for: Impact of the Potential m6A Modification Sites at the 3′UTR of Alfalfa Mosaic Virus RNA3 in the Viral Infection
Source: Viruses. 2022 Aug 4;14(8):1718. doi: 10.3390/v14081718 (PMC9414508; doi:10.3390/v14081718)

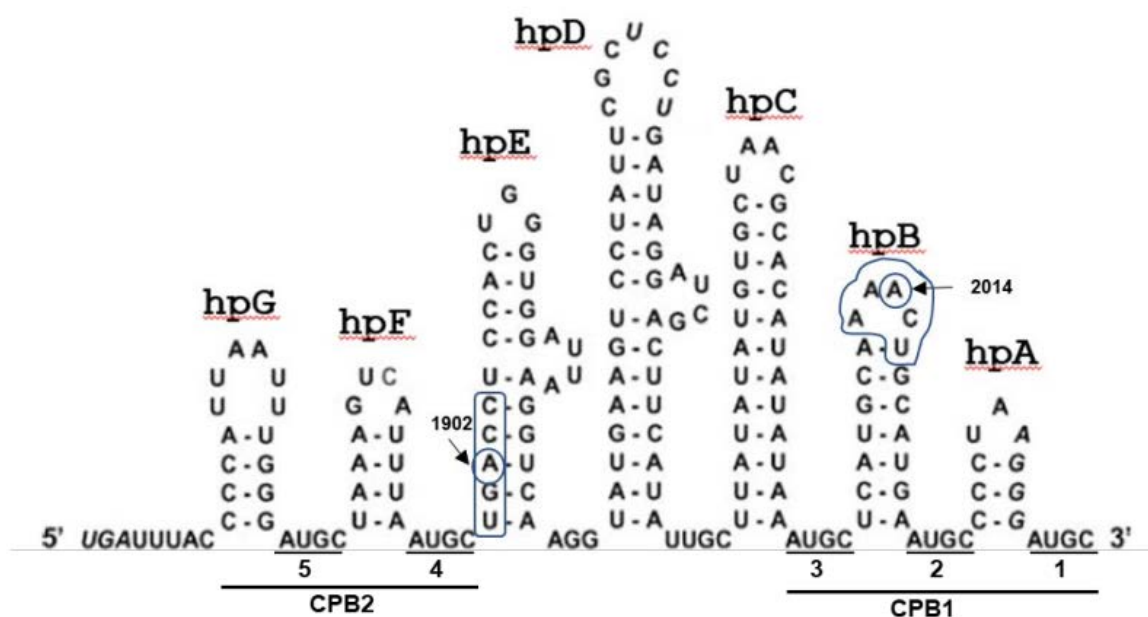

Supplementary Figure S1

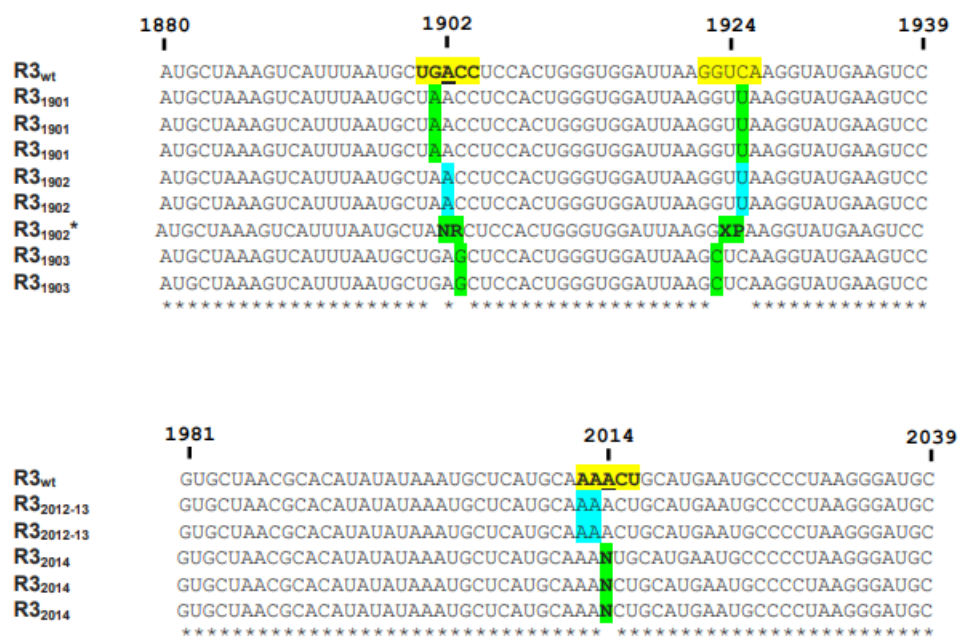

Supplementary Figure S2

Supplement: Supplementary file 1 [file viruses-14-01718-s001.zip › viruses-1851982-supplementary.pdf]
